# Seasonal Variation in Skin Cancer Diagnosis

**DOI:** 10.3389/fpubh.2016.00078

**Published:** 2016-04-28

**Authors:** Fortunato Bianconi, Giuseppe M. Masanotti, Arcangelo Liso, Francesco La Rosa, Emilio Duca, Fabrizio Stracci

**Affiliations:** ^1^Public Health Section, Department of Experimental Medicine, University of Perugia, Perugia, Italy; ^2^Umbrian Population Cancer Registry, Regional Government of Umbria, University of Perugia, Perugia, Italy; ^3^Department of Medicine and Surgery, University of Foggia, Foggia, Italy; ^4^Department of Health, Regional Government of Umbria, Perugia, Italy

**Keywords:** seasonal variation, skin cancers, melanoma, UV, cancer registry, access to care

## Abstract

**Purpose:**

Seasonality of skin cancer is well known, and it is influenced by a number of variables, such as exposure and personal characteristics, but also health service factors. We investigated the variations in the diagnosis melanoma skin cancer (MSC) and non-melanoma skin cancer (NMSC) during the year.

**Methods:**

We analyzed incident cases recorded in the Umbria Regional Cancer registry from 1994 to 2010 (1745 cases of MSC, 50% females, and 15,992 NMSC, 41% females). The Walter–Elwood test was used to assess seasonal effects. Relative risks were analyzed using negative binomial regression and splines.

**Results:**

Seasonality of MSC and NMSC was similar. Incidence peaks were observed in weeks 8, 24, and 43 (February, July, and October) and troughs in weeks 16, 32, 52, and 1 (August and December). Both NMSC and MSC cancers showed most elevated risks in autumn. A seasonal effect was present for trunk (*p* < 0.001) and absent for face cancers (*p* = 0.3).

**Conclusion:**

The observed pattern of diagnoses presumably depends on health service factors (e.g., organization of melanoma days, reduced access to care in August and during Christmas holidays) and personal factors (e.g., unclothing in the summer and delays in seeking care). High incidence rates in autumn could also in part depend on a late cancer progression effect of UV exposure. More efforts should be placed in order to guarantee uniform access to care through the year.

## Introduction

The seasonality of skin cancer occurrence has been confirmed in several studies (1–3). Seasonality has been explained with exposure, personal and health service factors, or a combination of the above. Several studies have suggested that chronic, low dose of ultraviolet radiation (UVR) may minimize the damage incurred from subsequent high-dose exposures; in fact, it has been demonstrated that suberythemal UVR is associated with stimulation of antioxidant enzymes (4–6), epidermal thickening, and enhanced DNA repair (1, 7, 8). Others have shown that repeated daily exposure of low-dose radiation can be demonstrated by the accumulation of epidermal DNA damage (9). The balance between the protection gained and damage incurred most likely depends on skin phototype and inherent DNA repair ability. Seasonality has been also referred to more intense exposure to UVR in the summer (3, 10, 11).

Some authors emphasized the influence of campaigns for early detection and more frequent detection of skin lesions due to unclothing in late spring and early summer (1–3, 12, 13).

In the Umbria, region of central Italy, non-melanoma skin cancer (MSC) is the most frequent cancer site. Incidence of melanoma is rather low, but an increasing trend is reported in both sexes. The standardized incidence rates (per 100,000 inhabitants) have increased, from 1994–1996 to 2008–2010, for MSC from 6.5 to 11.8 in males and from 6.4 to 11.5 in females, and from 65.5 to 103.8 in males and from 34.7 to 54.9 in females for non-melanoma skin cancer (NMSC) (14). Similar trends were reported for many western countries in the same period (15, 16). Explanations proposed for the observed trend include increased exposure to risk factors (e.g., sun exposure) and increasing skin examination for the early diagnosis of melanoma (17, 18). A number of statistical tests for the analysis of seasonal data, such as sinusoidal logistic regression, have been presented in the literature (19). This paper proposed an analysis that remove any constraint on seasonal pattern and also used weekly diagnosis data. Seasonal effects can contribute to define the role of risk factor exposures and diagnostic intensity in determining actual incidence patterns and trends. Therefore, we analyzed incident cases recorded in the Regional Cancer registry from 1994 to 2010. The primary goal of this paper is to investigate the influence of date of diagnosis on MSC and NMSC incidence.

## Cases and Methods

The Umbrian Cancer Registry (RTUP) started its activity in 1994 and covers a population of about 900,000 units, resident in the Umbria region, Italy. The population can be considered homogeneous for genetic background and social habits. This ensures that less confounding factors are present. Cases were collected in accordance with standard methods for cancer registries (20). Cancer type and site were coded using the ICD10 classification system (21). Cancer registry data were acquired and managed according to Italian laws and international quality rules for cancer registries. The in-house developed software guarantees also the anonymization of data management within the registry. The information system of the Umbria Cancer Registry (S.G.RTUP) was used for data management (22). Even if the microscopic verifications performed by regional dermatopathologists are regularly acquired, an additional investigation was performed for cases among all regional dermatologists to ensure completeness.

The incidence rate was calculated per 100,000 residents per year. The morphological verification was considered the date of diagnosis. The monthly incidence was calculated taking into account the exact numbers of days. Due to the relatively small number of cases, the incidence of melanoma was examined in total. Besides considering the overall distribution of cases by month of diagnosis, a comparison was made between two NMSC subsites: face (C44.0–C44.3 ICD10; 8700 cases) vs. trunk (C44.5 ICD10; 1800 cases). This is in order to explore the influence of clothing.

The Regional Central Reservations database, available from 2005, was used to extract skin-related specialist outpatient care codes. The selected procedures were removals of radical injury of the skin, removals or demolitions of local skin and subcutaneous tissue injury, and histopathological exams of skin or soft tissue from excisional biopsy. The weekly crude activity rate was evaluated as ratio between the number of access to the specialist outpatient and the population of the week. The statistical evaluation of seasonality on monthly data, adjusted for varying population at risk, was carried out by the Walter and Elwood test (23). The seasonality test and the goodness of fit test were produced. The chi-square test was used to compare distributions by gender.

To investigate the association between incidence and diagnosis month, we used negative binomial regression. This is used for count data when the counts are overdispersed, making a Poisson model inappropriate. Indeed, the heterogeneity parameter was significant in both the MSC (*p* < 0.001) and NMSC models (*p* < 0.001). The appropriate population at risk was included in the models (24). Nested models were compared using the likelihood ratio test.

A multivariate regression spline model was used to describe the correlation between incidence and month of diagnosis. Weekly data were used to obtain a better approximation of the spline function to the observed risks. Flexible models were adjusted by gender and sites (25). All tests were two sided, and results with *p* < 0.05 were considered significant. Analyses were carried out using Stata statistical software (26).

## Results

The site distribution and the number of examined cases are reported in Table 1. Importantly, 99.1% of MSC diagnoses and 99.4% of NMSC were of known morphology (i.e., microscopic verification was available). The subsite was specified in 89.9% of MSC and 85.8% of NMSC. Only two cases were known from death certificates only (DCO), and only six melanomas cases came from hospital discharge forms (SDO).

**Table 1:** **Skin cancer subsites**.

	MSC C43			NMSC C44		
	
	Males	Females	Total	Males	Females	Total
All subsites	870	875	1745	9475	6517	15,992
0.0 Skin of lip	0	2	2	290	195	485
0.1 Eyelid	2	6	8	434	390	824
0.2 External ear	12	12	24	896	172	1068
0.3 Other and unsp. Parts of face	61	87	148	3455	2868	6323
0.4 Scalp and neck	45	25	70	870	432	1302
0.5 Trunk	354	183	537	1099	701	1800
0.6 Upper limb	141	136	277	427	360	887
0.7 Lower limb	157	341	498	236	365	601
0.8 Overlapping	4	1	5	301	135	436
0.9 Skin, unspecified	94	82	176	1367	899	2266

In the period considered, 1745 cases of MSC (50% females) and 15,992 NMSC (41% females) were registered. The most frequent subsite for MSC was trunk (ICD 10 C43.5; 355 cases) and lower limb (ICD 10 C43.7; 342 cases) in males and females, respectively. Face (ICD 10 C44.3) was the most frequent NMSC subsite in both sexes (3313 cases in men and 2811 in women). All seasonality trends examined came out to be statically significant (Table 2).

**Table 2:** **Results of Walter–Elwood test for seasonality**.

Cancer type/site	Gender	*P*	Goodness of fit (*p*)
C43 MSC	M + F	0.0014	0.008
C44 NMSC	F	<0.001	<0.001
C44 NMSC	M	<0.001	<0.001
C44.0-0.3 face	M + F	<0.001	<0.001
C44.5 trunk	M + F	0.0013	<0.001

**Figure 1:**
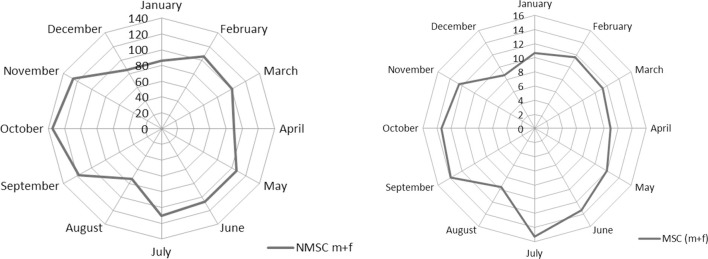
**Incidence of skin cancers by month of diagnosis (M + F)**.

The monthly distribution by gender for NMSC was not statistically different (chi-square test, *p* = 0.8). Comparing face NMSC with those of trunk, incidence differs for month of diagnosis (chi-square test, *p* < 0.001). Figure 1 pictures these seasonal trends. The monthly distribution of incidence of diagnosis of MSC and NMSC was similar and presented two peaks (the first one from February to July and the second peak from September to November). The lowest rates were recorded in August and in December–January.

**Figure 2:**
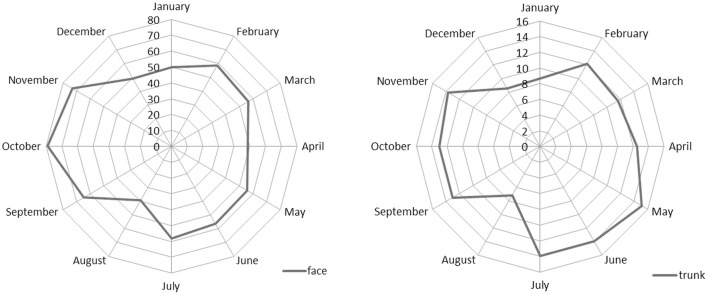
**Incidence of NMSC by month of diagnosis for face and trunk subsites (M + F)**.

Figure 2 shows the seasonality of face and trunk skin carcinomas by month of diagnosis. For both sites, the minimum was confirmed in August, but the number of trunk carcinoma cases was higher in spring while those of face were higher in autumn.

We fitted separate negative binomial regression models by cancer type for month of diagnosis (Table 3). This allows inspection and comparison of relative risks without constraint on seasonal pattern.

**Table 3:** **Negative binomial regression models for NMSC and MSC cancers by month of diagnosis**.

Month	NMSC (C44)				MSC (C43)			
	IRR	95% CI		*p*	IRR	95% CI		*p*
January	1.00	0.92	1.09	*0.99*	1.22	0.95	1.58	*0.12*
February	1.12	1.03	1.22	*0.007*	1.22	0.95	1.58	*0.12*
March	1.18	1.09	1.28	<*0.001*	1.31	1.02	1.68	*0.04*
April	1.01	0.93	1.10	*0.81*	1.21	0.94	1.57	*0.14*
May	1.25	1.15	1.36	<*0.001*	1.38	1.08	1.77	*0.01*
June	1.21	1.12	1.31	<*0.001*	1.50	1.18	1.92	*0.001*
July	1.29	1.19	1.40	<*0.001*	1.76	1.39	2.23	<*0.001*
August	0.86	0.79	0.94	*0.001*	1.11	0.86	1.44	*0.43*
September	1.34	1.23	1.45	<*0.001*	1.55	1.21	1.97	<*0.001*
October	1.58	1.46	1.70	<*0.001*	1.55	1.21	1.97	<*0.001*
November	1.43	1.32	1.54	<*0.001*	1.40	1.09	1.79	*0.008*
December^a^	Ref.				Ref.			
Gender								
Males	Ref.				Ref.			
Females	0.66	0.64	0.68	<*0.001*	0.94	0.86	1.03	*0.21*

*^a^Reference category is the month with the lowest incidence*.

We tested for a difference of incidence pattern by date of diagnosis. The negative binomial regression models including interaction terms between cancer type and month were compared to the nested models including main effects only. The results of the likelihood ratio tests were non-significant (*p* = 0.98).

**Figure 3:**
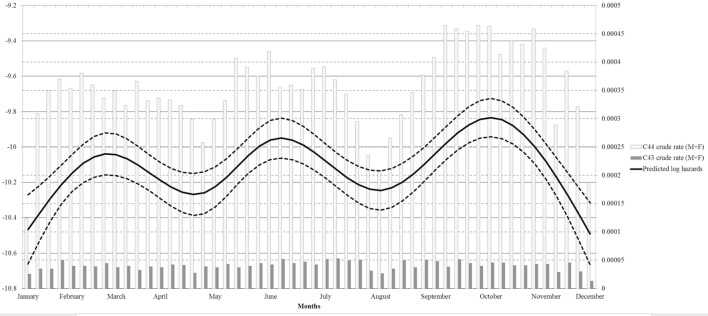
**Regression spline models for overall skin cancer risk adjusted by gender, site and population at risk by week of incidence**.

Restricted cubic splines were used in the negative binomial regression model to explore a continuous risk function for date of diagnosis (Figure 3). A single spline was used for MSC and NMSC cancers, assuming a common underling risk function. NMSC cases have more influence on the estimated functions than MSC due to the number of cases. Observed MSC and NMSC rates were plotted together with splines to provide a visual comparison of the continuous risk function to observed data. Notably, the result for date of diagnosis showed three separate high incidence peaks.

**Figure 4:**
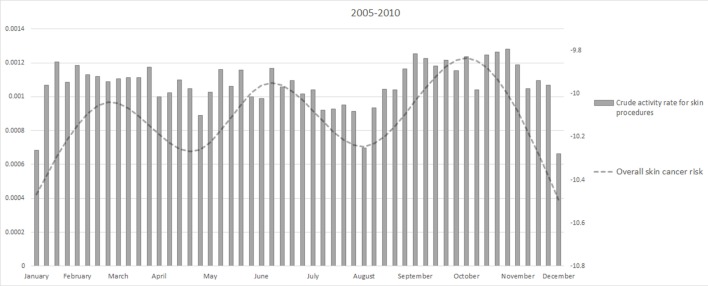
**Weekly activity rates from 2005 to 2010 and overall skin cancer risk**.

Figure 4 showed the weekly crude activity rate for skin-related procedures. The minimum of the activity rate were in August and Christmas period as for the overall skin cancer risk.

## Discussion

We observed that the registered incidence of skin cancers has a strong seasonal pattern. The incidence pattern supports the hypothesis that the seasonality of diagnosis is prevalently due to both personal factors (e.g., unclothing in the summer) and to access to care in different periods of the year.

Early diagnosis initiatives and summer unclothing can explain the incidence peak observed both for melanomas and carcinomas in the May–June period. In particular, in the Umbria region, a “skin cancer day” is carried out once a year on the first of May. Other initiatives including the “European Melanoma Day” take place also in late May or in June. As expected, these influences are less relevant for NMSC.

The difference in the seasonality of diagnoses observed for face and trunk NMSC probably reflects different exposure to inspection of body sites over the year. Moreover, the incidence of trunk NMSC is higher in the early summer than in autumn. The incidence pattern of trunk NMSC is similar to that of MSC and confirms the outcome reported by others for MSC (3–7).

The incidence peak in autumn could be explained by the delayed referral to medical examination of the skin lesions self-detected during August. UV exposure can also increase the pigmentation in melanocytic nevi that facilitates the detection of new or changing skin lesions (3, 27, 28). UV exposure causes both DNA damage and immunosuppression (29, 30). Chronic UV exposure is associated with skin cancer risk (31). Immunosuppression is also associated with skin cancer risk, particularly with NMSC (32). Indeed intense UV exposure during summer could even act as a late promoter with a short-term action (33). As the excess risk in autumn is much more evident for NMSC and it is present both for trunk and face subsites, the “late promoter hypothesis” clearly fits with the data for this cancer type, highlighting the need for further studies on this topic.

The strong decrease of diagnoses in August is likely due to the holiday period, relevant for both patients (delayed care seeking) and physicians (reduced access). Since in Italy, the paid leave regime offers 4 weeks’ vacation every year (34) and most people tend to take it in August, non-critical medical needs are often postponed (35). The same explanation (i.e., reduced access during Christmas holidays) applies to the low incidence rates that we observed in December and January. The effects of reduced access to care followed by an increase of diagnoses are evident from the weekly incidence rates and spline function (Figure 3). Previous studies, instead, analyzed only monthly data, therefore providing less informative results. Indeed average monthly risk (e.g., in January) can conceal a low incidence period followed by an incidence peak during the same month. Ideally, access to care should be uniformly warranted through all the year in order to avoid delayed diagnosis of skin cancer. In fact although the observed delays are unlikely to influence prognosis in most cases (36), we cannot exclude that selected patients would benefit from earlier diagnosis.

In general, the pattern of MSC incidence observed in this study is similar to that reported in another multicentric study performed by the Italian cancer registries (3).

Skin cancers pose a problem of completeness to cancer registries, as surgical treatment can be performed without hospitalization and histological verification can be carried out by a dermatopathologist, thereby not resulting in any cancer registry data sources (37). As a result, many cancer registries do not collect NMSC data at all because additional data source is needed to ensure completeness of registration (38, 39). Indeed, we performed an additional active search for cases among all regional dermatologists and regularly acquire archives from dermatopathologists to avoid incompleteness of registration. Therefore, we believe that population-based studies on NMSC, like ours, despite infrequent, do provide important information.

The present study has limitations. It is a retrospective study based on cancer registry data. Thus, the interpretation proposed for the observed seasonal pattern are speculative and should be confirmed in further analytic studies, even though we strengthened our observations with specific analyses (i.e., the weekly analyses showing strict correspondence between holidays period and incidence, comparison between cloth-covered sites and face skin cancer incidence, ecologic comparison with skin resection procedures).

In this paper, we applied the flexible parametric model as in our previous work (40). In principle, this model has the advantage to avoid constraint on seasonal pattern. However, a comparison of results from different methods in use to investigate seasonality would be interesting.

In conclusion, a seasonal effect was present in the diagnosis of MSC and NMSC; it is likely that pattern of incidence by month of diagnosis largely depends on health service factors (e.g., organization of melanoma days, reduced access to care in August and Christmas holidays) and personal factors (e.g., unclothing in summer and delay in seeking care). UV-associated immunosuppression and DNA damage due to summer UV exposure could be a late event in skin carcinogenesis, and it could contribute to explain the highest NMSC incidence peak observed in autumn.

## Author Contributions

FR designed the study. FS and FB coordinated the studies. FS drafted the paper and contributed to statistical analysis. FB performed data analysis. FB, AL, ED, and GM critically revised the manuscript. All authors read and approved the final manuscript.

## Conflict of Interest Statement

The authors declare that the research was conducted in the absence of any commercial or financial relationships that could be construed as a potential conflict of interest.
